# Monthly Summary

**Published:** 1894-06

**Authors:** 


### Monthly Summary.

SWALLOWED PLATES.—Three deaths in one month quite suggests the fact, that even the more mechanical side of our work should not be entrusted to the unskilled and ignorant. We confess we marvel not at these having died, but at the accident not being far more frequent than it is. The number of badly made Ill-fitting, worn-out dentures which are being worn is enormous. The first was the case of Andrew Henderson, who was employed by J. & G. Scott, oil merchants, Dobbie's Loan, St. Rollox.

Nothing definite is known as to how the accident occured, but on Monday, January 21st, Dr. Mackeller Dewar, Stirling Road, was called in, and on examination discovered that Henderson had swallowed his artificial teeth. He ordered his immediate removal to the Royal Infirmary where he was taken in an insensible condition. Every remedy was tried, but the man remained insensible and died on the following Saturday. The second was reported on January the 31st to the Liverpool coroner. The victim, Healey, aged 60, was a boatswain of a ship now in port; he had been seized with a fit of coughing, during which his artificial teeth became lodged in his windpipe. He was removed to the hospital, where an operation was performed, but the sufferer died. The third was a barman, who; some twelve months ago, accidentally swallowed his artificial teeth during a violent fit of coughing. He went to the London Hospital, but was not operated on, as the surgeons when sounding him were unable to find where the teeth had lodged. The patient afterward returned to work. On the 21st of January last he was suddenly seized with illness and died the same day. A *post-mortem* showed that the plate, containing four artificial teeth, had become imbedded in the upper part of the stomach on the right side, and around it ulceration had taken place; the immediate cause of death being due to hemorrhage.—*Dental Record.*

---

ALUMINUM PLATES.—I have given up vulcanite! Anyone who must have a rubber plate will never get it from me. I have done forever with the nasty, clumsy thing—excepting when I use enough of it to attach teeth to metal plates.

For several years I used cast aluminum plates, and, I confess, I had so many failures I returned to vulcanite; but now I am using aluminum exactly as I would use gold, for partial as well as for upper sets, and I have not only satisfaction myself, but I find my patients coming to me and wanting to get their vulcanite sets renewed for this lightest and pleasantest of all metals for the mouth. With care in striking up the plate I can make it yield to every obstruction. I make holes through it, and I secure perfect attachment.

"How do you do in a case of a single tooth, say a lateral incisor, where the space is so narrow you cannot get strength from a rubber attachment? What do you do in close bites where you cannot put any rubber at all?"

Simply this, I line and solder the tooth, and let a tail of gold plate extend beyond the line of the bite; I punch large holes through the tail, or solder a little loop or two to the end. I then rivet this to the aluminum, and there you are.—L. D. S. in *Dominion Dental Journal.*

---

KILLING AN ABSCESS.—Dr. J. D. Adair describes the method of using the kalium natrium (potassium sodium) preparation of Dr. Emil Schrier, and reports very satisfactory results in a number of cases. In one case he had vainly treated for six or eight weeks abscesses of three superior incisors, which were to receive Logan crowns. As the young lady had to return to her home in Baltimore, he concluded to make an application of the preparation and fill the roots without further delay. There was still pericemental tenderness with pus constantly present. The Logan crowns were put on at the final sitting. It was five months before he heard from the case and then he was greatly rejoiced to learn that the abscesses had given no further trouble and that the teeth were in every way comfortable.

It is now ten months since the crowns were put on and they are still perfectly satisfactory, having been heard from several times during the past five months.

---

AN IMPORTANT DECISION—THE LAW REGULATING DENTISTRY IN TENNESSEE TESTED.—Some time ago the State Board of Dental Examiners refused to register holders of diplomas of the Knoxville Dental College, that institution not having complied with the board's advanced standard of requirements for graduation. Suit was brought by the college in the name of M. B. Williams, one of its graduates, for a mandamus against the State Board to compel it to recognize his diploma. The case was tried before Judge Moon, at Chattanooga in February, who decided, according to the *Chattanooga Times* of March 3d, that "the powers of the State Board of Dental Examiners are at least quasi judicial, and that the courts are powerless by writ of mandamus to force any board of inferior jurisdiction, having judicial powers, to exercise those powers in any particular or designated way or manner; but if the power has once been exercised by said board, and its judicial determination made known, then if any other act by the board is to be performed—such as the mere issuance of a certificate—after the determination of the judicial question, such act is not judicial in its character, but purely ministerial, and the power of the court to force by writ of mandamus the performance of a ministerial act of this character cannot be questioned."

Thus it will be seen that the right of the board under our law to determine the reputability of a dental institution of learning is fully sustained, and its decision must stand unlcss overruled by the Supreme Court, to which tribunal an appeal was taken.—*Editorial in Headlight.*

---

AMERICAN DENTISTRY IN LONDON.—The object of this short paper is to throw light upon a subject of which comparatively little is known in America, and doubtless much chagrin has been experienced by the profession at large on account of the action of the General Medical Council in London in regard to the two dental schools recently disqualified for registration in Great Britian.

Yet when the facts have been clearly set before the profession, it will not be so surprising that such action was taken but rather that it was so long delayed. American dentistry in England is advertised most extensively and traded upon by those who practise, or pretend to practise it, simply because of the general admission in England of the superiority of it as compared with that practised by other nationalities. The easy credulity of the English public in this matter leads them to be duped by that which is called American, although those who practise under this title are not of that nationality or schooling. The consequence is, that the grossest maltreatment, to speak plainly, is perpetuated, both in supposed-to-be swell private practices as well as in the advertising "limited" companies, who carry on a trade in dentistry rather than a respectable practice. It is well known by the English dentists that this is a great wrong, not only to them, but to the people who are the victims of this avariciousness; they unscrupulously charge the exorbitant fee because it is "American dentistry," while the truth of the matter is, dentists of ability in the States are not guilty of performing operations such are classed here as American. As a result, the standard of American dentistry in London is anything but what we could wish, and, as seen by most English dentists, is far from being a credit to those who should be representatives of the profession, capable of upholding its high reputation and standard abroad. Why is this? naturally may be asked. The answer is not very hard to find, when it is known that there are several companies in London, with branches throughout England, who claim to be, and advertise as, Americans, and it is certain that many whom they employ are young men from the States. Every dentist who carries on a respectable practice will most emphatically condemn their methods. Some of the work done is not worthy of any American; yet fee enough is charged to command the best obtainable service from the hands of our experts. Their charges are excessive for what is really bad.

With this state of affairs going on for years, it is not surprising that our standard should be looked upon as no improvement upon that of the English. In private practices, there are many at present who for years worked for these firms, and finally established themselves when their time expired for which they were "bound out," for no one can secure a position who will not sign a contract that binds out of London for so many miles and for so many years. Some of these men go on with the same kind of work, and coin money. Even in the aristocratic West End (which one might think would be free from such) they are to be found. Some who never attended college put "Dr." on their door-plate, and "Doctor of Dental Surgery" appears, in so many words, on advertising cards, in defiance of law restricting them from practising in Great Britain. The are men who charge five hundred dollars (and it has been paid) for a full denture, gold base and rubber attachment, while if a tooth be a trifle off color, it is cut off and a crown put on at large expense. Many of these charlatans place large fillings over putrescent pulps, while the best is being paid for and supposed to be received. There are many working in this way, against whom the more learned and skilful have to compete, the latter in modest and professional manner, while the former class are obtrusive and gain the patronage of the wealthy and nobility, for the reason that, being Americans, better services are supposed necessarily to follow. What wonder that the standard is not being elevated! But some there are who are faithful and conscientious, and the English dentist is fair enough to acknowledge a good production from the land of the Stars and Stripes.

Graduates of American schools have practically no opportunity of securing good situations in England at the present time. A law has recently been passed by the General Medical Council forbidding dentists, under penalty of having their names removed from the register, to employ unregistered assistants. The only opening for the graduate is to take the examination for the degree of "L. D. S." (Ireland), "sine curriculo," in Dublin. Three harvard men have passed successfully, and are now registered. Let those who go to practise in London adhere to the principles they have been taught before leaving this country, and there will be a change for the better.

Let all diplomas be disqualified (as the British diplomas are) when graduates are involved with firms of the aforesaid description, and the day will not be far distant when a different state of affairs will be presented, and firms which bring discredit upon the fair name of American dentistry will have little excuse for their existence.—*International Dental Journal.*

DENTAL DOTS. BY V. D. BEACOCK.—Don't leave the cover off your vulcanizer when not in use.

Don't mix plaster to pour into a flask or impression cup, and when partially full mix a little salt or potassium with the remainder in the bowl, to make it set ; this will spoil all. Put it in just before commencing to pour.

Don't refuse to take a good dental journal. That community is to be more than pitied, whose dentist is too mean to read or subscribe for one or more dental journals.

Don't be too eager to take out six-year molars for children; you may live to regret it. Save all you can, except when common sense combined with dental knowledge otherwise dictates.

Don't use any copper amalgam in any of the teeth near the front of the mouth, no matter how small the cavity. If the patient is young it is sure to stain the tooth. I have seen several beautiful bicuspids ruined or otherwise disfigured for life with it.

Don't forget that kindness shown to children may, when they grow up, bring you many dollars.

Don't yield to the whim of every crank of a patient that may come along, by doing just whatever they may suggest, because they tell you some other dentist does it. Have an individuality of your own; be sure you are right, then stand by your convicitons.

Don't refuse to give a hint in the dental journal whenever you happen to have such a thing. Freely have ye received, now freely give.

Don't lower your prices, because some one tells you that Mr. So-and-so will do the same work for so much; it often happens that they are not telling the exact truth, but trying to beat you down—in other words, shopping.

Don't be in too great a hurry to find fault with some filling you may happen to find in the mouth not very well put in. It may be that the patient will calmly listen till you are through, then look you in the face, and say, "Why, sir, you put that filling in yourself!"

Don't make a funnel of your throat, to pour nauseous drugs and patent nostrums into your already over-jaded stomach, should you unfortunately be the victim of dyspepsia, hepatic or nephritic troubles, with their usual accompaniments, insomnia and neurasthenia, or any of the numerous diseases that dentists are so liable to. It would be much wiser to take a vacation, plenty of fresh-air exercise, using carefully prepared and easily digested food, with a judicious use of dumb-bells and Indian clubs. These latter will materially assist the metabolism and blood formation by accelerating the cell changes, and this too without the injurious effects of benumbing drugs, such as so-called tonics, nervines sedatives and drastic purgatives, which too often paralyze instead of strengthen the very organs they are supposed to aid. In fact, try and lead a life more in harmony with nature's law, and less vexing to both body and mind.—*Dominion Dental Journal.*

---

HOW TO GET CLEAN JOINTS. BY CHAS. SUTTON.— I have often read statements in the journals that the way to get clean joints is to grind closely, or to insert plaster, cement, etc., between the joints. Yet in spite of instructions, joints do come out with those reproachful dark lines which offend the eye. I do not pretend that I have made an original discovery, but I worked out the matter for myself, and I never have a dark joint, and this is the way I avoid it.

1. I never let wax get between the joints in preparing the set for the flask. I never melt the wax before or behind, where the blocks meet.

2. One of the last things I do when the case is waxed up, is to remove each block, one at a time, rub the joints on a piece of fine and clean sandpaper and replace them, taking care never to melt the wax where the blocks meet.

3. I then flask the set as usual, and when opening it, I avoid heating it so much that the wax will melt and run into the clean joints. I then *pick out* all the wax possible, especially in the vicinity where the blocks meet.

4. Now comes the secret. If you have not melted the wax into the joints before flasking, or when heating it to separate the flask, you have now perfectly clean joints. But you have to pour hot water into the case to melt out wax you cannot pick out, and when you do that, you just run the melted wax into those joints, and even if you boil for an hour, and pour gallons of boiling water into the case you can never cleanse them of foreign matter which not even boiling water will remove.

Now after you have picked out all the wax possible, just run into the joints thin plaster of Paris, and wait till it hardens. *Then* you may pour on your boiling water to remove the vestige of the wax; but neither the wax nor the water can get between the joints, and neither can the rubber. It is not the rubber that dirties the joints. It is the wax, and the foreign matter in the wax which you run into them when you think you are running it out.—*Dominion Dental Journal.*

---

DECAY OF TEETH COINCIDENT WITH ADVANCED CIVILIZATION.—There is reason to apprehend that unless some cataclysm occurs to arrest the progress of civilization, our decendents will be as toothless as Europtolemus, King of Cyprus, described by the historian Pliny as reduced to masticate his food with a structure of solid bone, in lieu of teeth. This, at any rate, is the inference to be deduced from the statistics recently published by order of the British Parliament, demonstrating the alarming small number of cases of sound dentition among the English. Of 4,000 children attending the London public schools, there were only 707 who had sound teeth; while during a period of three months 506 recruits were rejected by the medical department of the army for purely dental reasons. Of course, part of this state of affairs is due to neglect of digestion, and of the teeth themselves, a fact demonstrated by the statement that of all the girls who entered domestic service from the London public schools last year, five-sixths had never even heard of such a thing as a toothbrush—an assertion that has led the educational authorities to institute in many of the metropolitan schools what is now known as "toothbrush drill!" Decay of teeth has always attended the advance of civilization, and each barbaric invasion has been followed by a recovery of sound teeth in the Old World. Under the circumstances, it might be worth while to consider whether the repeal of the Geary law and the opening up of the United States to a Pacific invasion on the part of the Chinese, might not go far to improve the American jaw. For, according to medical experts, ours is in an immeasurably worse condition than that of the English, a fact probably due to our superior civilization.—*New York Tribune.*

---

TREATMENT OF BROKEN IMPRESSIONS.—That portion of the impression coming away with the tray is placed on a blotting pad, and the pieces as they are removed are placed by the side of the tray; those belonging to the right side of the mouth at the right of the tray, and those of the left to the left. This blotting pad, you will see, answers a threefold purpose: of a nice clean piece of paper on which to lay the impression, to keep the instrument bracket clean, and something on which to carry the impression to the laboratory; and it also assists in the hardening of the plaster by absorbing the moisture, so that in a few minutes it may be handled without fear of breaking in the process of putting together.

---

Dr. T. W. Brophy says: "I hold that any dentist educated within the schools of dentistry on the lines taught, is authorized under the laws of the State chartering the institution to practice in either department, and has equal rights to administer remedies and perform surgical operations with the general surgeon."

---

A new porcelain has been obtained by grinding asbestos to a fine powder and dissolving out all soluble matter with hydrochloric acid. The powder thus obtained is made into a paste with water, and baked for eighteen hours at 1,200 degrees in a furnace.

